# Serum Oxysterol Levels in Heart Failure With Preserved Ejection Fraction: A Prospective Case-Control Study

**DOI:** 10.7759/cureus.92947

**Published:** 2025-09-22

**Authors:** Musa Dagli, Murat Kerkütlüoğlu, Filiz Alkan Baylan, Hakan Gunes

**Affiliations:** 1 Cardiology, Akşehir Devlet Hastanesi, Konya, TUR; 2 Cardiology, Kahramanmaraş Sütçü İmam University, Kahramanmaras, TUR; 3 Biochemistry, Necmettin Erbakan Üniversitesi Tıp Fakültesi, Konya, TUR; 4 Cardiology, Health Sciences University, Faculty of Medicine, Izmir, TUR

**Keywords:** 7-ketocholesterol, biomarkers, heart failure with preserved ejection fraction, nt-probnp, oxysterols

## Abstract

Background: Heart failure with preserved ejection fraction (HFpEF) is a heterogeneous clinical syndrome with increasing prevalence and limited targeted therapeutic options. Oxysterols, i.e., oxidative derivatives of cholesterol, have been implicated in various cardiovascular pathologies through pro-apoptotic, pro-inflammatory, and cytotoxic mechanisms. However, their potential role in HFpEF pathophysiology remains unexplored.

Objective: This study aimed to investigate the relationship between serum oxysterol levels and HFpEF, and to evaluate their potential as novel biomarkers in this patient population.

Methods: In this prospective, single-center study, 101 participants were enrolled between September 27, 2022, and March 27, 2023. The study group consisted of 51 patients diagnosed with HFpEF, according to the current European Society of Cardiology (ESC) guidelines, while 50 age- and sex-matched individuals without HFpEF served as controls. Serum levels of 7-ketocholesterol, 25-hydroxycholesterol, and 7α,25-dihydroxycholesterol were measured using liquid chromatography-mass spectrometry (LC/MS). Clinical, biochemical, and echocardiographic parameters were recorded and compared between groups. Correlation analyses were performed to assess the relationship between oxysterol levels and other variables, including N-terminal pro-B-type natriuretic peptide (NT-proBNP) and echocardiographic measurements.

Results: Median serum levels of 7-ketocholesterol, 25-hydroxycholesterol, and 7α,25-dihydroxycholesterol were significantly higher in the HFpEF group compared to controls (p < 0.001 for all). Strong positive correlations were found between oxysterol levels and NT-proBNP concentrations (r = 0.778, r = 0.733, and r = 0.630, respectively; p < 0.001). Additionally, oxysterol levels showed weak positive correlations with body mass index and left atrial diameter. No significant associations were observed between oxysterol levels and comorbidities such as hypertension, diabetes mellitus, hyperlipidemia, or coronary artery disease.

Conclusion: This is the first study to demonstrate significantly elevated serum oxysterol levels in HFpEF patients compared to healthy controls. The strong correlation with NT-proBNP suggests that oxysterols may serve as potential biomarkers for HFpEF, reflecting underlying pathophysiological mechanisms involving inflammation and myocardial remodeling. Larger, multicenter studies are warranted to confirm these findings and explore their prognostic value.

## Introduction

Heart failure (HF) remains a major cause of morbidity and mortality worldwide, with its prevalence steadily increasing due to population aging and improved survival after acute cardiovascular events [1]. Traditionally, HF has been classified according to left ventricular ejection fraction (LVEF) into three distinct phenotypes: heart failure with reduced ejection fraction (HFrEF; LVEF <40%), heart failure with mildly reduced ejection fraction (HFmrEF; LVEF 41-49%), and heart failure with preserved ejection fraction (HFpEF; LVEF ≥50%) [2]. According to the 2021 European Society of Cardiology (ESC) guidelines, HFpEF is defined by the presence of typical symptoms and signs of HF, an LVEF of 50% or greater, elevated natriuretic peptide levels, and objective evidence of structural and/or functional cardiac abnormalities consistent with diastolic dysfunction and elevated filling pressures [3].

HFpEF accounts for more than half of all HF cases, and its prevalence is rising, particularly among elderly women and patients with comorbidities such as hypertension, obesity, and diabetes mellitus [4,5]. Despite its growing clinical impact, HFpEF remains challenging to diagnose and treat, largely due to its heterogeneous pathophysiology and the lack of effective, targeted therapies [6]. Current diagnostic algorithms, such as the H2FPEF and HFA-PEFF scores, have improved non-invasive evaluation; however, there is still a need for reliable biochemical markers that can aid in early diagnosis and risk stratification [7,8].

Oxysterols are oxygenated derivatives of cholesterol, formed either enzymatically via cytochrome P450-dependent pathways or through non-enzymatic oxidation processes [9]. They are highly lipophilic molecules capable of crossing cell membranes and have been implicated in various biological processes, including cholesterol homeostasis, inflammation, apoptosis, and immune regulation [10-12]. Pathologically, elevated oxysterol levels have been associated with atherosclerosis, neurodegenerative disorders, and chronic inflammatory conditions [13-15].

Given their pro-inflammatory and pro-apoptotic properties, oxysterols may play a role in the myocardial remodeling and endothelial dysfunction observed in HFpEF. However, to the best of our knowledge, no previous studies have investigated serum oxysterol concentrations in HFpEF patients. We hypothesize that circulating oxysterol levels are elevated in HFpEF and may correlate with established HF biomarkers such as N-terminal pro-B-type natriuretic peptide (NT-proBNP).

Therefore, the aim of this study was to compare serum levels of 7-ketocholesterol, 25-hydroxycholesterol, and 7α,25-dihydroxycholesterol between HFpEF patients and healthy controls, and to explore their potential associations with clinical, biochemical, and echocardiographic parameters.

## Materials and methods

Study design and population

This prospective, single-center, case-control study was conducted at the Department of Cardiology, Kahramanmaraş Sütçü İmam University Health Practice and Research Hospital, Turkey, between September 27, 2022, and March 27, 2023. The study protocol was approved by the institutional ethics committee, and written informed consent was obtained from all participants in accordance with the Declaration of Helsinki.

A total of 101 individuals were enrolled: 51 patients diagnosed with HFpEF (study group) and 50 age- and sex-matched healthy individuals without HFpEF (control group). HFpEF was diagnosed according to the 2021 ESC guidelines, requiring (1) clinical signs and/or symptoms of HF, (2) LVEF ≥50% on echocardiography, (3) evidence of diastolic dysfunction, and (4) NT-proBNP levels above the diagnostic threshold. NT-pro BNP levels have been determined as values above 125 pg/mL for patients with sinus rhythm or 365 pg/mL for patients with atrial fibrillation.

Inclusion criteria

Participants were eligible if they were aged ≥18 years, provided informed consent, had echocardiographic evidence of diastolic dysfunction, had signs and symptoms consistent with HF, demonstrated LVEF ≥50%, and had an H2FPEF score ≥5.

Exclusion criteria

Exclusion criteria included age <18 years, refusal to participate, LVEF <50%, severe valvular heart disease, chronic obstructive pulmonary disease, chronic connective tissue or autoimmune diseases (e.g., familial Mediterranean fever, rheumatoid arthritis, scleroderma, and gout), and use of corticosteroid medications.

Data collection

Demographic characteristics, anthropometric measurements, medical history (including hypertension, diabetes mellitus, hyperlipidemia, and coronary artery disease), and presenting symptoms were recorded. Blood pressure and body mass index (BMI) were measured at baseline.

Laboratory analysis

Fasting venous blood samples were collected from all participants. Routine laboratory measurements included complete blood count, serum electrolytes (sodium, potassium), fasting glucose, renal and liver function tests, lipid profile, albumin, and total protein. NT-proBNP was measured at the time of presentation.

For oxysterol analysis, 20 mL of venous blood was drawn into serum separator tubes, centrifuged at 4,000 rpm for 10 minutes, and aliquots were stored at −80°C until analysis. Serum levels of 7-ketocholesterol, 25-hydroxycholesterol, and 7α,25-dihydroxycholesterol were quantified using the Oxysterol Derivatization MaxSpec® Kit (Cayman Chemical, Ann Arbor, MI) and liquid chromatography-mass spectrometry (LC/MS) following the manufacturer’s protocol.

Echocardiographic assessment

Comprehensive transthoracic echocardiography was performed in the left lateral decubitus position using a GE Vivid E9 ultrasound system (GE Healthcare, Chicago, IL) equipped with a 5-1 MHz transducer by an experienced cardiologist blinded to clinical data. Standard two-dimensional, M-mode, and Doppler measurements were obtained according to the American Society of Echocardiography guidelines. Left atrial diameter (LAD), interventricular septal thickness (IVSD), posterior wall thickness (PWT), and left ventricular end-diastolic diameter (LVEDD) were measured.

Statistical analysis

Sample size estimation was based on prior studies examining oxidized cholesterol levels, assuming a power of 80% and α = 0.05, yielding a required minimum of 50 participants per group. Continuous variables were expressed as mean ± standard deviation (SD) or median and interquartile range (IQR), depending on normality assessed by the Kolmogorov-Smirnov test. Categorical variables were presented as frequencies and percentages. Intergroup comparisons were performed using an independent t-test or Mann-Whitney U test for continuous variables, and the chi-square test for categorical variables. Correlations were assessed using Pearson or Spearman correlation coefficients, as appropriate. A two-tailed p-value ≤ 0.05 was considered statistically significant. Statistical analyses were conducted using SPSS version 25.0 (IBM Corp., Armonk, NY).

## Results

Baseline characteristics

A total of 101 participants were included: 51 in the HFpEF group (Group 1) and 50 in the control group (Group 2). The overall cohort comprised 61 females (60.4%) and 40 males (39.6%). Mean age was slightly higher in the HFpEF group compared to controls (65.75 ± 10.48 vs. 62.24 ± 7.45 years), but this difference did not reach statistical significance (p = 0.056). The prevalence of hypertension, diabetes mellitus, hyperlipidemia, and coronary artery disease did not differ significantly between groups (all p > 0.05).

Atrial fibrillation (AF) was more frequent in the HFpEF group, though the difference was borderline non-significant (21.6% vs. 8.0%, p = 0.055). Baseline demographic and clinical characteristics of the study population are summarized in Table 1.

**Table 1:** Baseline demographic and clinical characteristics of the study population. HFpEF: heart failure with preserved ejection fraction.

Variable	HFpEF group (n = 51)	Control group (n = 50)	Test statistic	p-value
Age, years (mean ± SD)	65.75 ± 10.48	62.24 ± 7.45	t=1.94	0.056
Male sex, n (%)	18 (35.3%)	22 (44.0%)	χ²=0.80	0.371
Hypertension, n (%)	42 (82.4%)	42 (84.0%)	χ²=0.05	0.825
Diabetes mellitus, n (%)	18 (35.3%)	12 (24.0%)	χ²=1.54	0.214
Hyperlipidemia, n (%)	24 (47.1%)	26 (52.0%)	χ²=0.25	0.619
Coronary artery disease, n (%)	22 (43.1%)	17 (34.0%)	χ²=0.89	0.346
Atrial fibrillation, n (%)	11 (21.6%)	4 (8.0%)	χ²=3.65	0.055

Serum oxysterol levels

Median serum concentrations of all three oxysterols (7-ketocholesterol, 25-hydroxycholesterol, and 7α,25-dihydroxycholesterol) were significantly higher in the HFpEF group than in controls (all p < 0.001). Serum oxysterol levels in HFpEF and control groups are summarized in Table 2.

**Table 2:** Serum oxysterol levels in HFpEF and control groups. HFpEF: heart failure with preserved ejection fraction.

Parameter	HFpEF group, median (IQR)	Control group, median (IQR)	Test statistic	p-value
7-ketocholesterol (ng/mL)	41 (38–44)	23 (20.8–25.0)	U=210.0	<0.001
25-hydroxycholesterol (ng/mL)	1.50 (1.45–1.54)	1.23 (1.16–1.25)	U=205.0	<0.001
7α,25-dihydroxycholesterol (ng/mL)	0.009 (0.007–0.010)	0.005 (0.004–0.006)	U=215.0	<0.001

Oxysterol levels and comorbidities

When stratified by sex, hypertension, diabetes mellitus, hyperlipidemia, or coronary artery disease status, oxysterol levels showed no statistically significant differences (all p > 0.05).

Echocardiographic and laboratory parameters

Compared to controls, HFpEF patients had significantly higher NT-proBNP levels (296.0 (185.3-475.0) vs. 70.0 (70.0-103.2) pg/mL, p < 0.001), larger left atrial diameter (40.0 (35.0-43.0) vs. 35.0 (33.0-38.0) mm, p < 0.001), greater interventricular septal thickness (12.0 (11.0-13.0) vs. 10.0 (9.0-12.0) mm, p < 0.001), and greater posterior wall thickness (11.0 (11.0-12.0) vs. 9.0 (8.0-10.8) mm, p < 0.001). No significant differences were observed for LVEF, LVEDD, BMI, or other biochemical parameters (all p > 0.05). Echocardiographic measurements and laboratory parameters of the two groups are presented in Table 3.

**Table 3:** Echocardiographic and laboratory parameters in HFpEF and control groups. HFpEF: heart failure with preserved ejection fraction; LDL: low-density lipoprotein; LVDD: left ventricular end-diastolic diameter; NT-proBNP: N-terminal pro-B-type natriuretic peptide; HDL: high-density lipoprotein; AST: aspartate aminotransferase; ALT: alanine aminotransferase; LVEF: left ventricular ejection fraction; LA: left atrial; IVSD: interventricular septal thickness; PWT: posterior wall thickness.

Parameter	HFpEF group, median (IQR) or mean ± SD	Control group, median (IQR) or mean ± SD	Test statistic	p-value
Sodium (mEq/L)	139.94 ± 2.84	139.38 ± 2.11	t=1.12	0.263
Albumin (g/L)	43.29 ± 3.00	44.17 ± 3.73	t=1.29	0.199
LDL (mg/dL)	107.78 ± 35.81	110.98 ± 32.40	t=0.56	0.577
Total cholesterol (mg/dL)	185.53 ± 41.98	171.40 ± 41.35	t=1.70	0.092
Platelet count (/µL)	247000 ± 63506	265780 ± 61911	t=1.50	0.136
Hemoglobin (g/dL)	13.16 ± 1.62	13.74 ± 1.91	t=1.65	0.102
LVDD (mm)	47.57 ± 3.60	46.64 ± 2.98	t=1.41	0.161
BMI (kg/m²)	26.99 (24.52–29.72)	26.11 (24.71–26.98)	U=1180.0	0.710
Creatinine (mg/dL)	0.85 (0.68–1.02)	0.77 (0.68–0.96)	U=1170.0	0.336
NT-proBNP (pg/mL)	296.0 (185.3–475.0)	70.0 (70.0–103.2)	U=350.0	<0.001
Potassium (mEq/L)	4.5 (4.3–4.8)	4.5 (4.18–4.70)	U=1200.0	0.352
Glucose (mg/dL)	105.0 (91.0–136.0)	99.5 (92.5–125.7)	U=1190.0	0.265
Triglyceride (mg/dL)	167.0 (103.0–267.0)	133.5 (96.5–224.5)	U=1130.0	0.225
HDL (mg/dL)	47.0 (36.0–54.0)	40.5 (35.8–51.0)	U=1080.0	0.068
Total protein (g/L)	68.5 (65.0–73.0)	70.2 (69.0–73.0)	U=1090.0	0.125
AST (U/L)	18.0 (14.0–24.0)	20.5 (16.0–27.0)	U=1070.0	0.055
ALT (U/L)	18.7 (12.0–22.0)	21.2 (14.8–24.5)	U=1110.0	0.228
LVEF (%)	60.0 (55.0–60.0)	60.0 (58.8–60.0)	U=1150.0	0.187
LA diameter (mm)	40.0 (35.0–43.0)	35.0 (33.0–38.0)	U=650.0	<0.001
IVSD (mm)	12.0 (11.0–13.0)	10.0 (9.0–12.0)	U=640.0	<0.001
PWT (mm)	11.0 (11.0–12.0)	9.0 (8.0–10.8)	U=660.0	<0.001

Correlation analysis

Spearman correlation analysis revealed strong positive correlations between NT-proBNP levels and each oxysterol: 7-ketocholesterol (r = 0.778, p < 0.001); 25-hydroxycholesterol (r = 0.733, p < 0.001); 7α,25-dihydroxycholesterol (r = 0.630, p < 0.001).

Weak positive correlations were found between BMI and 7-ketocholesterol (r = 0.221, p = 0.026) and between left atrial diameter and all three oxysterols (r range: 0.336-0.410, all p < 0.01). Weak negative correlations were observed between 7-ketocholesterol and serum albumin, total protein, and alanine aminotransferase levels (all p < 0.05). Correlation analysis between oxysterol levels and clinical/laboratory parameters is presented in Table 4.

**Table 4:** Correlation analysis between oxysterol levels and clinical/laboratory parameters. NT-proBNP: N-terminal pro-B-type natriuretic peptide; HDL: high-density lipoprotein; LDL: low-density lipoprotein; AST: aspartate aminotransferase; ALT: alanine aminotransferase; LVDD: left ventricular end-diastolic diameter; LVEF: left ventricular ejection fraction; LA: left atrial.

Variable	7-ketocholesterol r (p-value)	Test statistic (t)	25-hydroxycholesterol r (p-value)	Test statistic (t)	7α,25-dihydroxycholesterol r (p-value)	Test statistic (t)
BMI	0.221 (0.026)	t≈2.28	0.116 (0.248)	t≈1.16	0.088 (0.381)	t≈0.89
Creatinine	0.058 (0.566)	t≈0.57	0.008 (0.937)	t≈0.08	0.061 (0.545)	t≈0.62
NT-proBNP	0.778 (<0.001)	t≈12.1	0.733 (<0.001)	t≈11.0	0.630 (<0.001)	t≈8.1
Sodium	0.136 (0.176)	t≈1.37	0.088 (0.380)	t≈0.89	0.049 (0.625)	t≈0.49
Potassium	0.093 (0.357)	t≈0.94	–0.011 (0.916)	t≈–0.11	0.067 (0.504)	t≈0.67
Glucose	0.036 (0.724)	t≈0.36	0.088 (0.382)	t≈0.89	–0.031 (0.760)	t≈–0.31
Albumin	–0.210 (0.036)	t≈–2.14	–0.196 (0.051)	t≈–1.98	–0.130 (0.198)	t≈–1.31
Triglyceride	0.079 (0.436)	t≈0.80	0.078 (0.444)	t≈0.79	0.062 (0.543)	t≈0.62
HDL	0.134 (0.184)	t≈1.36	0.144 (0.156)	t≈1.45	–0.011 (0.910)	t≈–0.11
Total cholesterol	0.118 (0.246)	t≈1.19	0.132 (0.195)	t≈1.33	0.092 (0.366)	t≈0.93
Total protein	–0.229 (0.023)	t≈–2.32	–0.059 (0.564)	t≈–0.59	–0.192 (0.057)	t≈–1.96
LDL	0.008 (0.935)	t≈0.08	0.029 (0.777)	t≈0.29	0.025 (0.806)	t≈0.25
ALT	–0.299 (0.002)	t≈–3.11	–0.195 (0.051)	t≈–1.97	–0.162 (0.105)	t≈–1.64
AST	–0.191 (0.055)	t≈–1.92	–0.192 (0.054)	t≈–1.93	–0.152 (0.129)	t≈–1.54
LVEF	–0.129 (0.199)	t≈–1.30	–0.042 (0.677)	t≈–0.43	–0.085 (0.396)	t≈–0.85
LA diameter	0.391 (<0.001)	t≈4.22	0.410 (<0.001)	t≈4.46	0.336 (0.001)	t≈3.45
LVDD	0.084 (0.402)	t≈0.85	0.092 (0.362)	t≈0.94	0.131 (0.192)	t≈1.32
Hemoglobin	–0.178 (0.076)	t≈–1.79	–0.128 (0.202)	t≈–1.29	–0.272 (0.006)	t≈–2.80
Platelet count	–0.095 (0.346)	t≈–0.97	–0.112 (0.266)	t≈–1.13	–0.011 (0.915)	

## Discussion

In this prospective case‑control study, we found that serum concentrations of 7‑ketocholesterol, 25‑hydroxycholesterol, and 7α,25‑dihydroxycholesterol were significantly higher in patients with HFpEF compared with controls. These oxysterols also correlated strongly with NT‑proBNP and modestly with LAD. Taken together with the recognized heterogeneity of HFpEF and its microvascular‑inflammatory paradigm, our data suggest oxysterols may index key biological pathways relevant to this syndrome [1,16,17].

Oxysterols and cardiovascular biology

Oxysterols are oxygenated cholesterol derivatives formed enzymatically or via non‑enzymatic oxidation. They regulate cholesterol homeostasis and immune signaling but can also promote inflammation, apoptosis, and cytotoxicity [9,10,13]. In vascular disease, oxysterols impair endothelial function and amplify oxidative stress, thereby fostering plaque inflammation and adverse remodeling [10]. These mechanisms conceptually intersect with HFpEF, where myocardial fibrosis, impaired relaxation, and increased passive stiffness are central [1,6,7,16].

Linking oxysterols to HFpEF pathophysiology

Contemporary models posit that comorbidities (hypertension, obesity, diabetes) trigger systemic inflammation and coronary microvascular endothelial dysfunction, leading to myocardial remodeling and stiffening in HFpEF [1,16,17]. Adipose tissue-derived immune and metabolic cues further sustain a low‑grade inflammatory milieu that may perpetuate diastolic dysfunction [18,19]. Within this framework, elevated oxysterols could reflect, and perhaps contribute to, endothelial injury, fibroblast activation, mitochondrial stress, and impaired calcium handling in cardiomyocytes, all of which have been implicated in diastolic dysfunction [6,7,16,20].

Association with NT‑proBNP and structural remodeling

NT‑proBNP is released in response to wall stress and is integral to HF diagnosis and risk stratification [1]. The strong correlations we observed between each oxysterol and NT‑proBNP suggest oxysterols may complement natriuretic peptides by capturing inflammatory‑metabolic stress alongside hemodynamic load. The positive association with LAD, an integrated marker of chronically elevated filling pressures, aligns with prior observations that atrial remodeling is tightly linked to HFpEF severity and outcomes [6]. Although oxysterols have been extensively studied in atherosclerosis and other cardiovascular contexts [10,21], to our knowledge, they have not previously been evaluated in HFpEF. Our data extend this literature by positioning oxysterols within the HFpEF ecosystem and by identifying 7‑ketocholesterol as the molecule showing the largest between‑group difference, consistent with its potent pro‑inflammatory and cytotoxic profile described elsewhere [21]. Moreover, the elevation of oxysterols despite similar BMI distributions implies disease‑specific metabolic dysregulation rather than a mere reflection of adiposity, concordant with phenotype‑based frameworks for HFpEF [17,22-24].

Clinical implications

Potential applications include (i) diagnostic support, especially when natriuretic peptide values are borderline; (ii) risk stratification, given associations with NT‑proBNP and LAD; and (iii) hypothesis‑generating therapeutic avenues targeting oxysterol biosynthesis or signaling. Any clinical implementation will require assay standardization and external validation.

Strengths and limitations

Strengths include prospective design, rigorous HFpEF adjudication per ESC guidance, and LC/MS quantification. Limitations include a single‑center setting, modest sample size, and cross‑sectional analyses that preclude causal inference. We also did not measure complementary inflammatory or oxidative biomarkers to triangulate the mechanism.

Future directions

Multicenter, longitudinal studies should test whether oxysterols add incremental diagnostic/prognostic information beyond natriuretic peptides and imaging and whether changes with therapy track clinical response. Mechanistic studies dissecting endothelial, fibroblast, and cardiomyocyte effects are warranted, as are interventional strategies modulating oxysterol pathways (e.g., CH25H/CYP27A1 axes) [25].

## Conclusions

In this prospective case-control study, we demonstrated for the first time that patients with HFpEF have significantly higher serum concentrations of 7-ketocholesterol, 25-hydroxycholesterol, and 7α,25-dihydroxycholesterol compared to healthy controls. These oxysterols showed strong positive correlations with NT-proBNP levels and moderate correlations with LAD, suggesting an association with both hemodynamic stress and structural cardiac remodeling.

Our findings indicate that oxysterols may play a role in the complex pathophysiology of HFpEF, potentially linking oxidative stress, inflammation, and myocardial fibrosis. The independence of these associations from traditional cardiovascular risk factors suggests that oxysterols may represent disease-specific biomarkers. If validated in larger, multicenter, longitudinal studies, oxysterol measurement could have clinical utility in the diagnosis, risk stratification, and therapeutic monitoring of HFpEF.
